# Comparative molecular analysis of chemolithoautotrophic bacterial diversity and community structure from coastal saline soils, Gujarat, India

**DOI:** 10.1186/1471-2180-12-150

**Published:** 2012-07-26

**Authors:** Basit Yousuf, Payal Sanadhya, Jitendra Keshri, Bhavanath Jha

**Affiliations:** 1 Discipline of Marine Biotechnology and Ecology, CSIR - Central Salt and Marine Chemicals Research Institute, G.B Marg, Bhavnagar, 364021, Gujarat, India

**Keywords:** *cbbL*, Clone libraries, 16S rRNA gene, RuBisCO, Barren soil

## Abstract

**Background:**

Soils harbour high diversity of obligate as well as facultative chemolithoautotrophic bacteria that contribute significantly to CO_2_ dynamics in soil. In this study, we used culture dependent and independent methods to assess the community structure and diversity of chemolithoautotrophs in agricultural and coastal barren saline soils (low and high salinity). We studied the composition and distribution of chemolithoautotrophs by means of functional marker gene *cbbL* encoding large subunit of ribulose-1,5-bisphosphate carboxylase/oxygenase and a phylogenetic marker 16S rRNA gene. The *cbbL* form IA and IC genes associated with carbon fixation were analyzed to gain insight into metabolic potential of chemolithoautotrophs in three soil types of coastal ecosystems which had a very different salt load and sulphur content.

**Results:**

In *cbbL* libraries, the *cbbL* form IA was retrieved only from high saline soil whereas form IC was found in all three soil types. The form IC *cbbL* was also amplified from bacterial isolates obtained from all soil types. A number of novel monophyletic lineages affiliated with form IA and IC phylogenetic trees were found. These were distantly related to the known *cbbL* sequences from agroecosystem, volcanic ashes and marine environments. In 16S rRNA clone libraries, the agricultural soil was dominated by chemolithoautotrophs (*Betaproteobacteria*) whereas photoautotrophic *Chloroflexi* and sulphide oxidizers dominated saline ecosystems. Environmental specificity was apparently visible at both higher taxonomic levels (phylum) and lower taxonomic levels (genus and species). The differentiation in community structure and diversity in three soil ecosystems was supported by LIBSHUFF (*P* = 0.001) and UniFrac.

**Conclusion:**

This study may provide fundamentally new insights into the role of chemolithoautotrophic and photoautotrophic bacterial diversity in biochemical carbon cycling in barren saline soils. The bacterial communities varied greatly among the three sites, probably because of differences in salinity, carbon and sulphur contents. The *cbbL* form IA-containing sulphide-oxidizing chemolithotrophs were found only in high saline soil clone library, thus giving the indication of sulphide availability in this soil ecosystem. This is the first comparative study of the community structure and diversity of chemolithoautotrophic bacteria in coastal agricultural and saline barren soils using functional (*cbbL*) and phylogenetic (16S rDNA) marker genes.

## Background

Chemolithoautotrophic bacteria utilize inorganic compounds as electron donors for growth. They are subdivided into two main groups based on their electron donors: Obligate lithotrophs including hydrogen-, sulphide-, sulphur-, metal-, ammonia-, nitrite- oxidizing bacteria and facultative lithotrophs such as CO-oxidizing bacteria [1]. Chemolithoautotrophic soil microorganisms contribute significantly in sequestration of the green house gas CO_2_ which helps in climate sustainability and assimilate CO_2_ mainly by Calvin-Benson-Bassham (CBB) pathway. However, some chemolithotrophs such as *Epsilonproteobacteria* have been reported to use the reductive tricarboxylic acid cycle [2]. The crucial enzyme of the CBB cycle is ribulose-1,5-bisphosphate carboxylase/oxygenase (RuBisCO) which occurs in four forms [3]. Form I RuBisCO found in higher plants, algae, *Cyanobacteria* and chemolithoautotrophs, is by far the most abundant enzyme in the world [4]. It is a bifunctional enzyme capable of fixing either CO_2_ or O_2_. It is commonly found in cytoplasm, but a number of bacteria package much of the enzyme into polyhedral organelles, the carboxysomes. These carboxysomes enhance CO_2_ fixation. This enzyme is climate resilient and consists of 8 large and 8 small subunits. Form I is considered to be evolved from form II, which consists of only large subunits [5]. *Archaea* contain a separate class of RuBisCO termed as form III [6,7]. Form IV has been found in *Bacillus subtilis*[8], *Chlorobium tepidum*[9] and *Archaeoglobus fulgidus*[10]. Form III and IV are referred as RuBisCO like proteins.

The large subunit of form I RuBisCO is encoded by *cbbL*-gene [11]. The form I RuBisCO is essentially found in two major forms, green like and red like, which show differences in their amino acid compositions [12]. The green like RuBisCO is divided into two types, IA and IB. Form IA is found in *Alpha*-, *Beta*- and *Gammaproteobacteria* and is phylogenetically allied to form IB which occurs in the chloroplasts of terrestrial plants, green algae and *Cyanobacteria*[12]. The red like RuBisCO is also divided into two relatively close forms, IC and ID. Form IC is found in *Alpha*- and *Betaproteobacteria* and many non green algae carry form ID [12]. Form IA genes are harboured by obligate and some facultative chemolithotrophs which utilize either inorganic or organic substrates [1]. However, there are some exceptions such as *Hydrogenophaga pseudoflava*, oxidizing CO and hydrogen but does not oxidize reduced sulphur species [13]. In contrast, form IC *cbbL* occurs in manganese-, CO- and hydrogen-oxidizing facultative chemolithotrophic bacteria that potentially use heterotrophic substrate as carbon sources. A distinct form of IC *cbbL* sequences are also reported in a group of ammonia-oxidizing *Nitrosospira* species [14].

The phylogenetic relationships of specific functional bacterial groups by use of 16S rRNA gene and a corresponding functional marker gene such as *nifHamoA* and *dsrAB* have been previously studied [15-18]. In this study we used 16S rRNA gene and a functional marker gene *cbbL* for determining phylogenetic relationships of chemolithoautotrophs. The phylogenetic affiliations based on *cbbL* gene are incongruent with 16S rRNA gene phylogeny due to horizontal gene transfer of the *cbbL*-gene. Nevertheless, the *cbbL*-gene seems to be useful for studying evolution and diversity of autotrophic organisms. This discrepancy in nature of RuBisCO phylogeny is only evident at higher taxonomic levels and has negligible apparent affect at lower taxonomic levels [19]. To date, molecular ecological studies based on RuBisCO genes are mostly restricted to aquatic systems [17,20-23] with relatively few analysis devoted to chemolithotrophs in soil [14,24] and fewer from extreme terrestrial systems [25,26]. Thus to gain an insight into specific biochemical pathways and evolutionary relationships, *cbbL* and 16S rRNA gene sequences were studied together in chemolithoautotrophs from coastal saline ecosystem.

In this study we report the diversity, community structure and phylogenetic affiliation of chemolithoautotrophic bacteria in two contrasting soil ecosystems i.e. agricultural soil and coastal barren saline soils using both culture dependent and independent methods. DNA was extracted from bacterial isolates as well as soil samples, *cbbL* (form IA & form IC) and 16S rRNA gene clone libraries were constructed and analyzed. The *cbbL* form IC sequences were most diverse in agricultural system while form IA was found only in one saline sample (SS2) which reflects the possible availability of sulphide in saline soil. This is the first comprehensive study on chemolithoautotrophs from coastal saline soil.

## Results

The three soils showed variations in water content, pH, salinity, organic carbon, nitrogen and sulphur contents (Table 1). The agricultural soil (AS) had electrolytic conductivity (EC) of 0.12 dS m^-1^ and pH 7.09 whereas the EC and pH of saline soils (SS1 & SS2) were 3.8 dS m^-1^, 8.3 and 7.1 dS m^-1^, and pH 8.0. Total carbon level varied with high content in agricultural soil (2.65%) and low content in saline soils SS1 (1.27%) and SS2 (1.38%). The nitrogen content was high in agricultural soil while sulphur concentration was high in saline soil SS2. DNA extraction from soil samples, PCR amplification and gene library construction were carried out in duplicate (per site). A comparison of sequences from each site (within transects) revealed that the libraries displayed 90-93% similarity with each other. This was well supported by weighted UniFrac environmental clustering analysis which indicated that the bacterial communities within sites were not significantly differentiated (UniFrac *P* = 0.5 for AS, 0.9 for SS1 and 0.9 for SS2) in both *cbbL* and 16S rRNA clone libraries. One of the clone libraries from each sample has been further analyzed.

**Table 1:** Physico-chemical properties of agricultural soil (AS) and saline soils (SS1 & SS2)

**Site**	**EC (dS m**^-1^**)**^1^	**pH**	**TC**^2^**(%)**	**TIC**^3^**(%)**	**TOC**^4^**(%)**	**TN**^5^**(%)**	**S**^6^**(%)**
AS	0.12	7.09	2.65	1.6	1.04	0.14	0.016
SS1	3.8	8.3	1.27	0.83	0.44	0.09	0.11
SS2	7.1	8.0	1.38	0.78	0.61	0.09	0.28

^1^ Electrolytic conductivity.

^2^ Total carbon.

^3^ Total inorganic carbon.

^4^ Total organic carbon.

^5^ Total nitrogen.

^6^ Sulphur.

### *CbbL* clone libraries (Form IC & IA)

**Figure 1:**
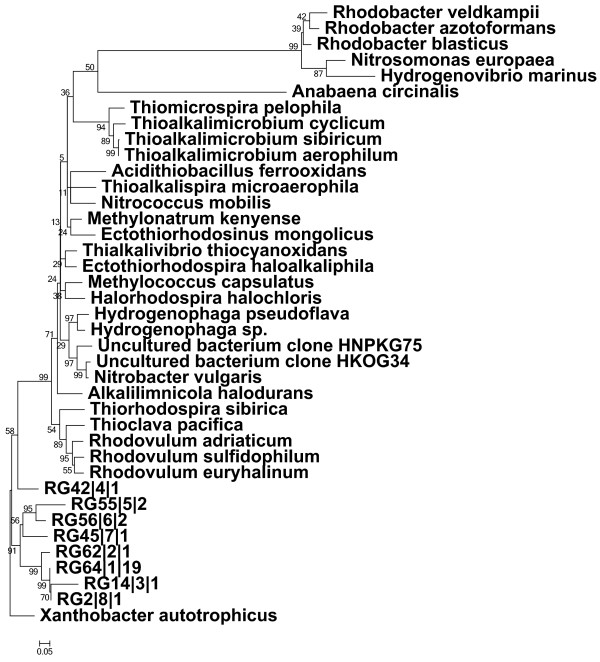
**Phylogenetic analysis of green like*cbbL*clones.** Neighbour-joining tree (Jukes–Cantor correction) was constructed from saline soil (SS2) clone library partial *cbbL* (form IA) nucleic acid sequences (phylotypes) with closely related *cbbL*-gene sequences from known organisms and environmental clones. Clone sequences of form IA *cbbL* sequence are coded as ‘RG’. One representative phylotype is shown followed by phylotype number and the number of clones within each phylotype is shown at the end. One thousand bootstrap analyses were performed and percentages are shown at nodes. The scale bar indicates 0.05 substitutions per site. The red-like *cbbL* sequence of *Xanthobacter autotrophicus* was used as outgroup for tree calculations.

*CbbL* clone sequences were grouped into OTUs based on a cut-off of 95% sequence similarity. Totals of 141, 99 and 103 form IC *cbbL* clone sequences were obtained from agricultural (AS) and two saline (SS1 & SS2) soils and termed BS, HS, and RS respectively. Overall, the red like clone sequences yielded 58, 32 and 40 unique phylotypes for AS, SS1 & SS2 clone libraries respectively. Heatmap (Additional file 1: Figure S1) generated by Mothur program depicts the relative abundance of these phylotypes within respective clone libraries. In spite of repeated attempts to amplify and clone PCR products, only 28 partial form IA clone sequences were obtained from the saline soil (SS2), termed “RG clones”, and could be grouped into 8 OTUs (Figure 1). Comparisons with the NCBI database by BLAST searches revealed that these OTUs were only distantly related to the known green-like *cbbL* sequences (Figure 1).

### Phylogenetic affiliation of RuBisCO genes

**Figure 2:**
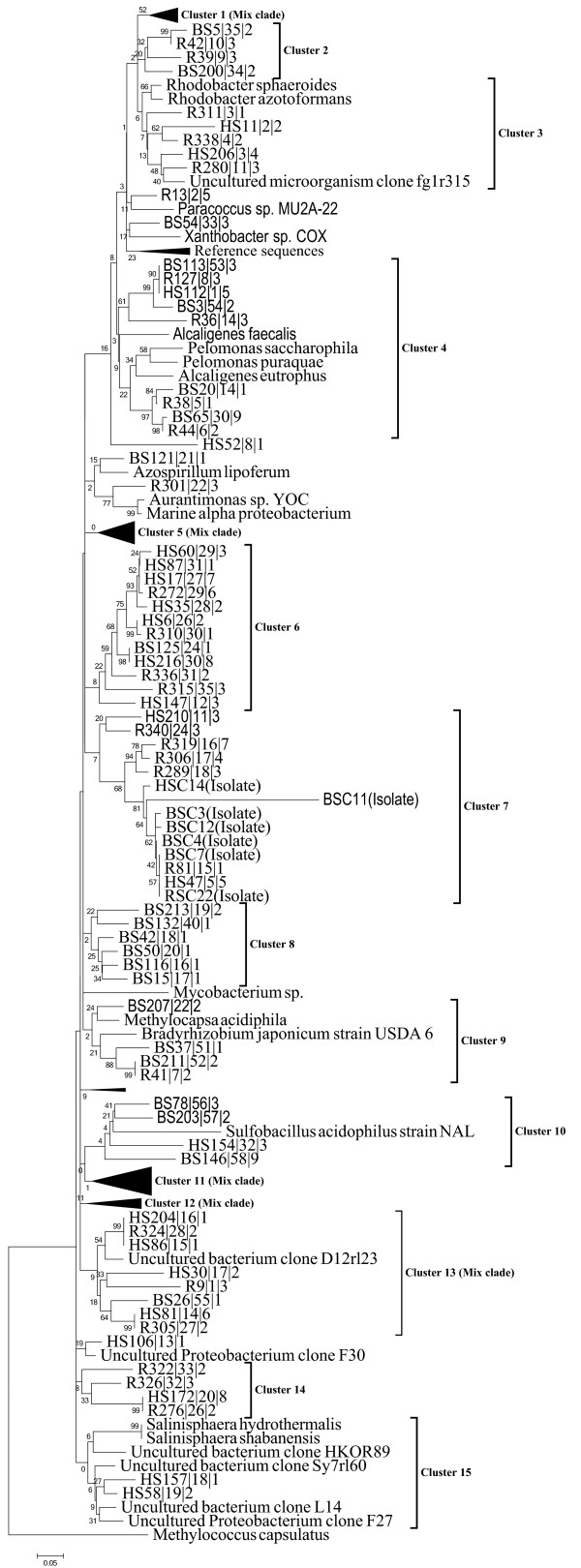
**Phylogenetic analysis of red like*cbbL*clones.** A composite neighbour joining tree (Jukes-Cantor correction) was constructed from aligned nucleotide sequences (phylotypes) of form IC *cbbL*-gene obtained from agricultural soil ‘AS’ and barren saline soils ‘SS1 & SS2’ with closely related *cbbL*-gene sequences from known organisms and environmental clones. Bootstrap values are shown as percentages of 1000 bootstrap replicates. The bar indicates 5% estimated sequence divergence. One representative phylotype is shown followed by phylotype number and the number of clones within each phylotype is shown at the end. Clone sequences from this study are coded as ‘BS’ (AS), ‘HS’ (SS1) and ‘R’ (SS2). The *cbbL*-gene sequences of the isolates from this study are denoted as ‘BSC’, ‘HSC’ and ‘RSC’ from AS, SS1 and SS2 respectively. The green-like *cbbL*-gene sequence of *Methylococcus capsulatus* was used as outgroup for tree calculations.

The phylogenetic trees were constructed by neighbour joining method using Jukes-Cantor correction. A composite phylogenetic tree was generated from selected nucleotide sequences of form IC *cbbL* genes from all three soil samples and bacterial isolates (Figure 2). Separate trees for AS and SS1 & SS2 were also generated from aligned nucleotide sequences of form IC *cbbL* genes (Additional file 2: Figure S2a and Additional file 3: Figure S2b). In the composite tree, majority of the phylotypes (60%) from different soil types did not cluster close to the *cbbL* sequences of known autotrophs. The sequences of cluster 2 (4 OTUs), cluster 6 (12 OTUs), cluster 7 (5 OTUs, 7 cultured isolates), cluster 8 (6 OTUs), cluster 13 (8 OTUs) and cluster 14 (4 OTUs) formed novel monophyletic groups not affiliated to known *cbbL* gene containing bacteria. Some of the clone sequences clustered with *cbbL* sequences from known lithotrophs. OTUs from AS soil were grouped into one site specific cluster (cluster 8). The phylotypes from saline soils were closely clustered within cluster 3, cluster 6, cluster 7, cluster 14 and cluster 15. The remaining clusters (1, 2, 4, 5, 9, 10, 11, 12 & 13) displayed random distribution of agricultural and saline soil OTUs, containing sequences from all three soil samples. The largest clade of the composite tree, cluster 11 (24 OTUs, 50 clones) comprised sequences having ubiquitous distribution in all three clone libraries (Figure 2), and was affiliated to *Rhizobium leguminosarum*.

In the phylogenetic tree constructed from the phylotypes of agroecosystem clone library, fifty eight OTUs could be classified into nine clusters with the largest clade (cluster 1) constituting 28% of clone library. Cluster 1 (14 OTUs, 40 sequences), cluster 2 (8, 17) cluster 3 (8, 12), cluster 4 (10, 17), cluster 5 (1, 1), cluster 6 (5, 17), cluster 7 (6, 15), cluster 8 (4, 10) and cluster 9 (5 cultured isolates) were grouped together in AS phylogenetic tree (Additional file 2: Figure S2a). Cluster 3 and 4 included reference sequence from *Bradyrhizobium japonicum, Rhizobium leguminosarum, Alcaligenes, Pelomonas, Paracoccus* and *Ochrobactrum anthropi*. The sequences of cluster 1 and 8 formed novel monophyletic groups without showing any affiliation with known *cbbL* gene containing organisms and constitute the majority of clones. The phylotype BS146 and cluster 9 (cultured isolates) constitute a branching lineage directly originating from the root not allied with any known organism. Two phylotypes BS203 and BS78 were related to *Sulfobacillus acidophilus* and formed a separate cluster with *Mycobacterium*.

In the phylogenetic tree constructed from the phylotypes of saline soil clone libraries, seventy two OTUs could be assigned to eight clusters, largest cluster being clade 1 constituting 17% of clone libraries (Additional file 3: Figure S2b). The OTUs were phylogenetically placed with different groups of autotrophic *Alpha-, Beta-* and *Gammaproteobacteria* which are abundant in soils. Most of the OTUs were not closely affiliated (<93% nucleotide similarity) with RuBisCO sequences in the database, however, some of them were clearly related to a variety of clone sequences reported from differently managed agricultural systems, volcanic deposits, marine environments, contaminated aquifers, deltaic mobile sediments, arid and grassland soils. In the phylogenetic tree from saline soils, OTUs from cluster 3 (9 OTUs and 32 clones), cluster 5 (12, 32), cluster 6 (3, 13), cluster 7 (6, 15) and cluster 8 (2, 6) grouped with *cbbL* sequences of known cultured organisms like *Rhodopseudomonas palustris, Oligotropha carboxidovorans, Nitrosospira, Rhizobium leguminosarum, Salinisphaera, Alcaligenes, Pelomonas, Paracoccus, Rhodobacter, Agrobacterium tumefaciens, Sinorhizobium fredii* and *Ochrobactrum anthropi* (79-88%). The *cbbL* sequences in the cluster 4 (8, 20) were grouped with *Aurantimonas* bacterium (4 OTUs), *Methylocapsa acidiphila* (one OTU), *Bradyrhizobium japonicum* (one OTU) and *Azospirillum lipoferum* (one OTU). Some sequences in the cluster 5 displayed sequence homology with *Nitrosospira.* Phylotype HS154 was distantly related with *Sulfobacillus acidophilus* and *Mycobacterium*. Cluster 1 (12, 35, 2 cultured isolates) showed a high intra cluster similarity not affiliated with any other known RuBisCO sequence and formed a monophyletic lineage with *cbbL* sequences of the cultured isolates (HSC14, RSC22) obtained from these soil samples. The phylotype R13 from saline soil constituted a distinct branching lineage not affiliated with any known *cbbL* containing cultured representative.

The form IA *cbbL* genes were amplified only from high saline soil (SS2). The phylogenetic analysis (Figure 1) revealed that the 8 phylotypes (28 clones) were not closely associated with known sulphide, ammonia oxidizers or other taxa and formed one separate monophyletic cluster. Furthermore, the form IA clone sequence RG42 was divergent from other form IA gene sequences.

### 16S rRNA clone library and phylogenetic analysis

**Figure 3:**
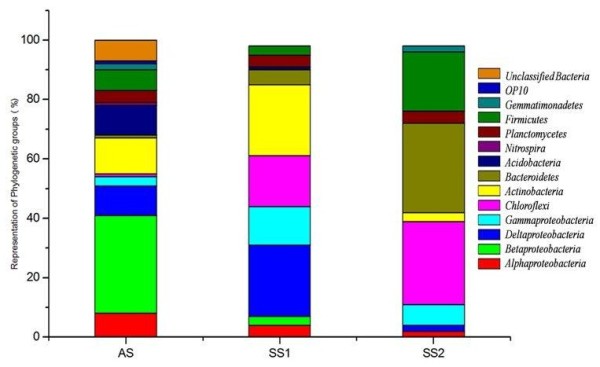
**Taxonomic distribution of different bacterial phylogenetic groups in agricultural soil (AS) and saline soils (SS1, SS2).** Analysis of amplified 16S rRNA gene sequences was done in comparison with the RDP II database (match length >1200 nucleotides). The percentages of the phylogenetically classified sequences are plotted on y-axis.

Total 329 16S rRNA gene clone sequences were retrieved from three soil samples. The RDP classifier was used to assign 16S rRNA gene sequences to the phylogenetic groups (Figure 3). Totally 227 OTUs were identified among the 329 clones in the combined data set. Comparative abundance of these OTUs was illustrated by heatmap (Additional file 1: Figure S1) generated by Mothur. A total of 147 clone sequences were analyzed from the agricultural soil (AS), which generated 109 unique OTUs that grouped within ten bacterial phyla*- Proteobacteria* (*Alpha, Beta, Gamma,* and *Delta*)*, Acidobacteria, Actinobacteria, Bacteroidetes, Chloroflexi, Cyanobacteria, Firmicutes, Gemmatimonadetes, Nitrospira* and *Planctomycetes*. A total of 97 and 85 gene sequences were analyzed from saline soils (SS1 & SS2) which generated 55 and 63 unique OTUs respectively. These OTUs grouped into different bacterial phyla as described above except *Cyanobacteria* and *Nitrospira*. The phylogenetic trees showing the taxonomic assignment of phylotypes to different bacterial groups were constructed from the three soil clone libraries (data not shown).

The detailed affiliation of different phylotypes with their closest neighbour in database is presented in Additional file 4: Table S1. The majority of phylotypes that belong to *Alphaproteobacteria* were from AS clone library. These OTUs were related (85-99%) to *Rhizobiales, Sphingomonadales* and *Rhodospirillales* while six OTUs from SS1 & SS2 libraries showed affiliation (89-99%) to *Rhodobacterales, Rhizobiales* and *Rhodospirillales*. A cluster of 25 sequences from AS clone library (7 OTUs), which contributes 58.7% of the total AS *Betaproteobacterial* population were related (87-99%) to *Limnobacter thiooxidans* from family *Burkholderiaceae*, formed one of its largest cluster. The only SS1 OTU HSS79 showed 97% similarity to uncultured *Betaproteobacteria* whereas no OTU was observed in SS2 clone library.

The 22 OTUs (4 from AS and 18 from SS1 & SS2 clone libraries) were related to different species of uncultured *Gammaproteobacteria.* Most of the SS1 & SS2 clone sequences were related to cultured bacteria like *Salinisphaeraceae* bacterium, *Methylohalomonas lacus,* sulphur-oxidizing bacterium and *Marinobacter* species*.* The presence of sulphur-oxidizing and *Marinobacter* bacteria in saline soils may suggest the presence of sulphur in these saline environments. These saline soils indeed contain sulphur (Table 1). *Deltaproteobacterial* OTUs from SS1 & SS2 clone libraries formed a tight cluster with deep sea bacterium, uncultured *Deltaproteobacteria* and *Marinobacterium.*

OTUs belonging to photoautotrophic *Cyanobacteria* and chemoautotrophic nitrifying *Nitrospira* were found only in AS clone library. Two phylotypes BSS159 and BSS49 were related (91%) to *Cyanobacteria* and uncultured *Nitrospira*, respectively and more may be present as rarefaction curves did not reached saturation, although started to level off. The photoautotrophic *Chloroflexi* related sequences were mostly from SS1 & SS2 clone libraries within the families *Caldilineaceae, Sphaerobacteraceae* and *Anaerolineaceae.* One OTU RS187 had 88% homology with *Sphaerobacter thermophilus*, no other OTUs were more than 91% similar to that of any described organism (Additional file 4: Table S1). There were only two OTUs from AS clone library which showed affiliation (>92%) to uncultured *Chloroflexi*. van der Meer *et al*. (2005) [27] suggested that *Cyanobacteria* and *Chloroflexi* utilize different spectra of light, and CO_2_ from the atmosphere for photosynthesis.

*Firmicutes* related sequences were found mostly in AS and SS2 clone library. One phylotype RS190 was affiliated with *Bacillus polygoni* (95%) a moderately halophilic, non-motile, obligate alkaliphile isolated from indigo balls. Two OTUs RS39 and RS147 showed close relationship with *Halanaerobiales* from which one phylotype was distantly related (84%) to *Halothermothrix orenii* a halophilic, thermophilic fermentative anaerobic bacterium [28].

The *Bacteroidetes* sequences were abundant in the SS2 clone library (Additional file 4: Table S1). Two phylotypes (RS23, RS17) were related to *Salinimicrobium catena* isolated from sediments of oil fields in the South China Sea [29] within *Flavobacteriaceae.* The *Acidobacteria* group was dominant in the AS clone library and the sequences were related (88-99%) to uncultured *Solibacter* isolated from hydrocarbon contaminated soils [30], and uncultured *Acidobacteria* isolated from the heavy metal contaminated soils [31]. No phylotype from SS2 was found related to this group. *Planctomycetes* group was represented by twelve OTUs (13 sequences), four from each soil sample. The OTUs from SS1 & SS2 clone libraries were related to uncultured marine bacteria and *Planctomyces maris* (Additional file 4: Table S1).

The *Actinobacterial* clones from AS clone library were related (93-99%) to *MicromonosporaArthrobacter globiformisStreptomyces* and *Rubrobacter radiotolerans*. Eleven OTUs from SS1 & SS2 clone libraries clustered with uncultured *Actinobacteria, Amycolatopsis* and *Nitriliruptor alkaliphilus*, a haloalkaliphilic actinobacterium from soda lake capable of growth on aliphatic nitriles [32]. Overall eight OTUs, six from AS and two from SS2 clone library were related (89-95%) to the uncultured *Gemmatimonadetes* bacterium. No OTU was found affiliated to the *Gemmatimonadetes* group in SS1 clone library. Three OTUs from AS clone library were related to the uncultured phylum *OP10*.

### Phylogenetic analysis of *cbbL* positive bacterial isolates

From a total of 22 bacterial isolates seven were positive for form IC *cbbL* genes. The positive isolates were analyzed for further study. The *cbbL*-gene sequences of the isolates from this study were denoted as ‘BSC’, ‘HSC’ and ‘RSC’ from AS, SS1 and SS2 soil samples, respectively. The nucleotide similarities of *cbbL* sequences retrieved from the bacterial isolates were distantly related (77-85%) to known *cbbL* sequences. The 16S rRNA gene sequences of the isolates from this study were denoted as ‘BSCS’ (AS), ‘HSCS’ (SS1) and ‘RSCS’ (SS2). A neighbour joining tree (Additional file 5: Figure S3) was constructed from 16S rRNA gene sequences of the bacterial isolates harbouring *cbbL* form IC gene. All seven *cbbL* positive bacterial isolates grouped with *Bacillus* species. Four isolates, one from each saline soil and two from agricultural soil were related to the *Bacillus firmus*. Two isolates from AS showed a very high homology (99%) with *B. vireti* whereas one isolate was related (99%) to *B. horikoshii*. Apparently, only a very limited diversity could be isolated using the single AT-medium under aerobic conditions without ascorbate.

### Diversity indices and community structure of *cbbL*/16S rRNA gene libraries

The parametric (Shannon and Simpson’s diversity indices) and non parametric (Chao and ACE) diversity indices (Table 2) indicated that the diversity of *cbbL* and 16S rRNA gene clone libraries of AS differed from SS1 and SS2 soil clone libraries. In 16S rRNA gene libraries the shared OTUs between three soils increased significantly on decreasing the similarity cut-off. This pattern was also evident from the *cbbL*-gene sequence analysis. The rarefaction curve of form IC *cbbL*-gene sequences (distance = 0.05) did not reach an asymptote in AS clone library whereas rarefaction curves reached near saturation in SS1 & SS2 clone libraries (Additional file 6: Figure S4a). Rarefaction curves for 16S rRNA gene libraries reached near an asymptote for SS1 and SS2 saline soils at the estimated phylum level 80% (Additional file 6: Figure S4b). The agricultural soil gene library represented non asymptotic curve at phylum level (80%) as well as at the species level (98%) similarity cut-off. In general, the bacterial species richness in agricultural soil was greater than saline soils as indicated by the inclines in rarefaction curves.

**Table 2:** **Biodiversity and predicted richness of the*cbbL*and 16S rRNA gene sequences**

**Genes**	**No of clones**	**Coverage (%)**	**Evenness(J)**	**Shannon Weiner (H)**	**Simpson (1-D)**	**Sobs**^1^**(OTU)**	**Chao**	**ACE**	**No of Singletons**
*cbbL* form IC									
AS	141	83	0.92	3.7	0.98	58	71.8	87.2	24
SS1	99	91	0.92	3.2	0.96	32	34.3	37.6	8
SS2	103	91	0.94	3.5	0.97	40	43.6	43.8	9
*cbbL* form IA									
SS2	28	82	0.58	1.2	0.55	8	11.3	16.8	5
16S rRNA									
AS	147	33	0.92	4.3	0.98	109	584.3	4626.3	98
SS1	97	56	0.92	3.7	0.97	55	206.5	553.5	41
SS2	85	36	0.93	3.9	0.97	63	311.5	1278.9	53

^1^OTUs for *cbbL*-gene clone libraries were determined at a 0.05 distance cut-off and OTUs for 16S rRNA clone libraries were determined at a 0.02 cut-off using the MOTHUR program. The Coverage, Shannon-Weiner (H), Simpson (1-D), Evenness (J) indices and Chao & ACE richness estimators were calculated using the OTU data.

**Figure 4:**
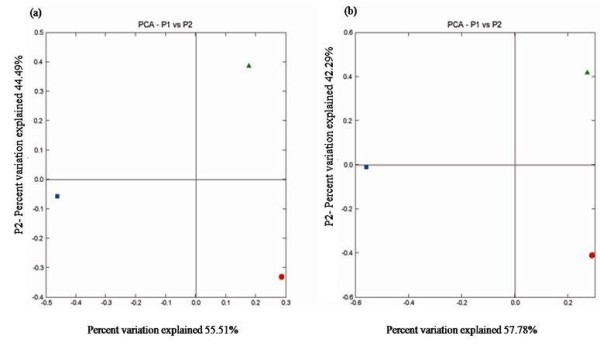
**UniFrac PCA of*cbbL*and 16S rRNA clone libraries.** The ordination plots for the first two dimensions to show the relationship between agricultural and the saline soils for (**a**) *cbbL* and (**b**) 16S rRNA gene assemblages. Agricultural soil (AS) is represented by square and saline soils is represented by diamond (SS1) and circle (SS2). Each axis indicates the fraction of the variance in the data that the axis accounts for.

The lack of substantial overlap between soil clone libraries suggests that bacterial communities were unique to each soil habitat. This observation was statistically supported by using LIBSHUFF (*P* = 0.001 for the average pairwise comparison for three sites), suggested that the bacterial communities retrieved from *cbbL* and 16S rRNA analysis were significantly different from one another across the sites (Additional file 7: Figure S5). The difference between homologous and heterologous coverage curves was determined by distribution of ΔC as a function of evolutionary distance. Our results showed significant difference between libraries with considerable ΔC values at D below 0.2 (Additional file 7: Figure S5). This result suggests that differences were between closely related sequences. This conclusion was also supported by the phylogenetic trees in which the sequences from different clone libraries often group near each other but were rarely identical. We employed phylogenetic tree based comparisons, the UniFrac metric, and phylogenetic *P*-test to *cbbL* and 16S rRNA clone libraries. Weighted UniFrac environmental clustering analysis indicated that the assemblages of bacterial sequences in agricultural soil were marginally differentiated from those found in the saline soils (UniFrac *P* < = 0.03). To determine whether the samples clustered in two dimensional spaces, PCA was applied to UniFrac metric. The ordination diagram (Figure 4a) of *cbbL* clone libraries revealed that strongest variation in the data set was between agricultural soil and saline soils as they were separated on first axis of ordination diagram, which explains high percentage of total variation (55.51%). In case of 16S rRNA gene clone libraries*,* the first axis separated agricultural and saline soils which explain total community variability (57.78%) among three sample sites (Figure 4b).

## Discussion

The study of microbial diversity is crucial for the understanding of structure, function, and evolution of biological communities in order to effectively link community structure and function. We constructed multiple clone libraries for each gene (*cbbL* form IC, IA and 16S rRNA) from agricultural and saline soils, which were further analyzed. Form IC was highly diverse in all clone libraries while form IA could only be amplified from the high saline soil (SS2) clone library (Table 3). This is in accordance with the previous work reported by Nanba *et al*. (2004), Tolli & King (2005) and Selesi *et al*. (2005) [14,24,33]. They also found form IC *cbbL* sequences almost exclusively dominant in various terrestrial (agroecosystem, pine forest) systems and noted that form IA was less diverse than form IC.

**Table 3:** **Oligonucleotide primers used for PCR amplification of*cbbL*and 16S rRNA genes**

**Primer**	**Position(nt)**	**Primer sequence(5′-3′)**	**Reference**	**PCR amplification**^1^		
				**AS**	**SS1**	**SS2**
*cbbL*R1F	634-651	AAGGAYGACGAGAACATC	Selesi *et al*., 2005 [24]	+	+	+
*cbbL*R1R	1435-1454	TCGGTCGGSGTGTAGTTGAA				
*cbbL*G1F	397-416	GGCAACGTGTTCGGSTTCAA	Selesi *et al*., 2005 [24]	-	-	-
*cbbL*G1R	1413-1433	TTGATCTCTTTCCACGTTTCC				
RubIgF	571-590	GAYTTCACCAARGAYGAYGA	Spiridonova *et al*., 2004 [34]	-	-	+
RubIgR	1363-1382	TCRAACTTGATYTCYTTCCA				
27 F	27-46	AGAGTTTGATCMTGGCTCAG	Lane, 1991 [35]	+	+	+
1492 R	1471-1492	TACGGYTACCTTGTTACGACT				

^1^Positive PCR amplification (+), no PCR amplification (−) for the targeted primers in three soil samples.

This study targeted functional and phylogenetic markers together in order to reveal the metabolic potentialities of the chemolithoautotrophic bacteria at three different soil habitats. Comparison of microbial populations between different soil habitats includes diversity estimation based on the expected number of OTUs. For the determination of relative diversity captured from each soil sample, both parametric (Shannon and Simpson’s diversity indices) and non parametric (Chao and ACE) diversity indices were calculated. Each diversity index is associated with the specific biases. The Shannon index takes into account consistency of species abundance in OTUs, while the Simpson’s index is sensitive to abundant OTUs [36]. Chao richness is based on singletons and doubletons [37], while ACE is based on the distribution of abundant (≥10) and rare (≤10) species. A higher bacterial diversity was observed in the agricultural soil in comparison to the saline barren soils as revealed by Shannon and Simpson diversity indices and other non parametric indices (Table 2). This suggests that the autotrophic bacterial distribution is likely to respond to different environmental variables such as pH, salinity, organic carbon and nitrogen concentrations etc. and the dominant populations are selected in response to changes in these variables. The soil carbon and sulphur content appears to be the major determinants of microbial community structure and function in the soil samples. But it is difficult to ascertain which particular environmental variables are driving the observed pattern of biological diversity as many of the soil and environmental characteristics are interrelated. Environmental stability is important to the development and maintenance of biodiversity [38]. Stable environments are thought to support a higher degree of organisation, more complex food webs, more niches, and ultimately more species [39]. Our data is in agreement with these assumptions as barren coastal saline soil ecosystem does not remain stable because of tidal influx thus representing less diverse ecosystem as compared to more stable agroecosystem.

LIBSHUFF analysis of *cbbL* and 16S rRNA clone libraries verified a large degree of variability in agricultural and saline soils in all pairs of reciprocal comparisons. The differential community structure and membership in agricultural soil as compared to the saline soils were in agreement with our expectations. A change in the community composition with increase in salinity was evident at the phylum level. Microorganisms adapt to the altered salinity or they are replaced by microorganisms adapted to the changed conditions [40]. The replacement mechanism appears to operate at the phylum level, as changes of major groups were observed with increased salinity. However, at micro diversity level the gradual evolution and adaptation might take place (Figure 3) [41]. The analysis of OTUs shared between three soils revealed that bacterial communities from both the saline soils were more similar than that of agriculture soil as depicted by the overlap in Venn diagram of *cbbL* and 16S rRNA gene clone libraries between the communities at species level cut-off (Additional file 8: Figure S6). Multiple clones from the saline soil libraries clustered together and were closely related as revealed by Lineage specific analysis in UniFrac.

Form IC sequences were affiliated to *Alpha-, Beta-* and *Gammaproteobacteria* for which chemolithotrophy and/or sulphur metabolism is a major mode of energy generation. In the composite tree, molecular phylogenetic analysis of *cbbL* clone libraries demonstrated the presence of six different novel monophyletic lineages of *cbbL* harbouring chemolithoautotrophic bacteria residing in the agroecosystem and saline soil clone libraries (Figure 2). These *cbbL* genes had a low sequence similarity with *cbbL*-types from known organisms, which indicates the sources of these *cbbL* genes may be yet unknown and uncultured autotrophic bacteria. The *cbbL* sequences fall into 15 clusters; one cluster AS site specific, five clusters SS1 & SS2 site specific and nine clusters having *cbbL*-gene sequences obtained from all three sampling sites. The ubiquitous distribution of majority of the phylotypes (nine mix clades) in the agroecosystem and saline soil clone libraries suggest a possible large scale distribution of several closely related chemolithotrophs. However, the possibility of high degree of sequence conservation and horizontal gene transfer in RuBisCO gene has limited the inference about taxonomic identity of closely related clones [19]. The saline soils phylotypes were assigned to some recognized genera like *Nitrosospira, Paracoccus, RhodobacterSalinisphaera,* and many uncultured clones from differently managed agricultural systems, contaminated aquifers and deltaic mobile sediments. These sequences from saline soil clone libraries mostly belong to *Alpha-* and *Betaproteobacteria*. The other important members of chemolithoautotrophic community in saline soils were *Gammaproteobacterial* autotrophs which were found predominantly in saline soil. The *Gammaproteobacteria* are previously known to be dominated by obligate haloalkaliphiles, for example, cluster 15 has sequences related to the genus *Salinisphaera* which are halophilic, aerobic, facultatively chemolithoautotrophic bacteria oxidizing CO and thiosulphate [42]. Some sequences from saline soil were related to nitrifying photoautotrophic purple non sulphur bacterium *Rhodobacter* and denitrifying bacterium *Paracoccus*. One phylotype was related to the *Aurantimonas* bacterium which is facultative lithotrophic marine manganese oxidizing bacteria. The agricultural clone library phylotypes tightly clustered with different genera of *Alphaproteobacteria* and *Betaproteobacteria* like *Rhizobium, Bradyrhizobium, Xanthobacter, Beijerinckia, Sulfobacillus, Oligotropha* and uncultured bacterial clones from grassland soils [26] and arid soils. *Bradyrhizobium japonicum* is a facultative chemolithoautotroph and utilizes thiosulphate and H_2_ as an electron donor and CO_2_ as a carbon source [43]. In cluster 10 three phylotypes from AS and one from SS1 clone libraries were related to *Sulfobacillus acidophilus* (sulphide oxidizing bacteria) and *Mycobacterium* of phylum *Actinobacteria*. The form IC sequences in the agroecosystem library suggest a dominance of the facultative chemolithotrophic community like CO & hydrogen oxidizing, nitrogen fixing, and plant growth promoting bacteria. None of the sequences cluster closely with *Nitrosospira* clade, this may be due to the low abundance of ammonia oxidizers or PCR and DNA extraction biases. The agricultural soil being sulphur poor system does not significantly support the sulphur/sulphide oxidizing bacterial populations.

All the *cbbL* positive cultured isolates were closely related to different species of the genus *Bacillus.* A RuBisCO like protein (RLP), form IV RuBisCO was previously isolated and studied from *B. subtilis* and this RLP is involved in methionine pathway [44]. However, the form IC gene sequences from the isolates in this study are different from the form IV RLP gene *ykrW* of *B. subtilis.* Recent studies suggested that RLP and photosynthetic RuBisCO might have evolved from the same ancestral protein [45]. Presence of form IC genes in cultured *Bacillus* sp. was also reported by Selesi *et al.* (2005) [24]. But a clear proof, whether the *Bacillus* isolates are completely functional autotrophs, is not yet documented. Further analysis of evolutionary and functional relationships between RLPs and RuBisCO may explain the presence of these form IC genes in *Bacillus.*

The amplification of form IA *cbbL* genes in SS2 soil only by Spiridonova *et al.* (2004) [34] primers proves the primer selectivity bias. This could be supported by suppression of autotrophic bacterial growth by readily available carbon sources in case of agricultural soil [46,47]. Role of variation in other physico-chemical properties between different sites on form IA gene diversity also cannot be underestimated. In our study, most of form IA clone sequences did not cluster closely with the sequences from known sulphide oxidizing lithotrophs. This reflects that limited attention has been paid to the role of lithoautotrophs in coastal saline environments. Further isolation attempts using a variety of different media are necessary to isolate this mostly unrevealed diversity in these soils.

The 16S rRNA gene sequence analysis was aimed at providing further information about the total bacterial communities. If 16S rRNA gene sequences were more than 95% similar to that of known autotrophic bacteria that genus is recognized for some form of chemolithoautotrophy and photoautotrophy [48]. Sequences inferred to be from potential CO_2_ fixing chemolithotrophs from groups *Alpha-* and *Betaproteobacteria* were highly abundant in the agricultural soil whereas *Gammaproteobacteria, Deltaproteobacteria, Actinobacteria* and phototrophic *Chloroflexi* dominated saline soils. Among the *Betaproteobacteria* two OTUs (22 clones, AS) were very closely related to *Limnobacter thiooxidans* (99%), which can grow chemolithoheterotrophically by oxidation of thiosulphate to sulphate [49]. One phylotype was related (96%) to *Azohydromonas australica*- nitrogen and hydrogen utilizing bacteria [50]. Two OTUs from AS clone library belonged to the phylum *Nitrospira*, which are facultative chemolithoautotrophic nitrite oxidizing bacteria [51]. We also obtained one phylotype from AS clone library related to the *Cyanobacteria,* an oxygen evolving and chlorophyll containing photosynthetic bacterium. Our agricultural clone libraries did not suggest an abundance of nitrite-oxidizing *Nitrospira* and phototrophic *Cyanobacteria* in the soil, a few sequences were identified and more may be present because the rarefaction curves (Additional file 6: Figure S4b) did not reach an asymptote. The *Gammaproteobacteria* sequences in SS2 clone library were related to the phototrophic *Ectothiorhodospira*, an alkaliphilic and halophilic purple sulphur bacterium from soda lake [52]. The phylotype HSS148 was distantly related (88%) to the chemolithotroph *Thioalkalivibrio*, which oxidizes sulphide or thiosulphate with molecular oxygen. Nine OTUs from *Deltaproteobacteria* (SS1 clone library) fell into the order *Desulfovibrionales*, which oxidizes reduced sulphur compounds using a variety of electron acceptors. The light penetration through soil is minimal [53] however, the presence of *Chloroflexi* (filamentous anoxygenic phototrophs) in deeper soil layers (0 to 10 cm) was observed in all three soil samples. This can be justified by the fact that light of higher wavelengths has the potential to penetrate deeper into the soil [54], which are used by the *Chloroflexi*[27].

Many of the sequences from saline soils have been previously reported from different saline environments, and the current study added significantly to the genetic pool of extreme and normal terrestrial habitats. The diversity and composition of the bacterial community along the three soil habitats varied with increase in salinity (Figure 3). The change in the relative proportion of the *Betaproteobacteria* from agricultural to saline soil habitats is particularly more apparent. Wu *et al*. (2006) [40] reported that with increasing salinity, the relative abundance of *Betaproteobacteria* decreases while that of *Alpha-* and *Gammaproteobacteria* increases. The low salinity of agricultural soil may, therefore, explain the high *Betaproteobacteria* diversity in AS clone library. The relative abundance of the *Alpha*- and *Gammaproteobacteria* does not show any systematic change*. Alphaproteobacteria* were abundant in AS clone library and *Gammaproteobacteria* were abundant in the saline soil clone libraries (Figure 3). Hansel *et al*. (2003) [55] showed the inverse relationship between carbon availability and abundance of *Acidobacteria*. However, the *Acidobacteria* group in our study did not show such relationship. The *Acidobacteria* sequences retrieved from the poor carbon saline soils was only 0.5%, but they were abundant (14.6%) in agricultural soil. The possible explanation for this may be the difference in other physico-chemical properties of the soils. For example, an inverse relationship between *Acidobacteria* diversity abundance and soil pH has also been previously reported [56]. *Firmicutes* related sequences were more abundant in saline soils in comparison to the agricultural soil. This predominance of *Firmicutes* related sequences in saline soils is consistent with the previous studies. For example, the *Firmicutes* are absent in a number of hypersaline environments [57,58] but abundant in low salinity environments such as deep sea sediments [59]. *Chloroflexi* sequences were present at each of the three sites, however, they were most abundant at barren saline soils. *Chloroflexi* groups are the potential phototrophs and were abundant in barren soils [25]. This can be speculated as the saline soils provide open areas of exposed soil that can favour diverse photoautotrophic microbes [60,61].

## Conclusions

The four *cbbL* libraries studied in this work demonstrated the presence of highly diversified and partially unique *cbbL* sequences, which could belong to the possibly yet unknown potent CO_2_-fixing bacteria. The *cbbL* form IA gene containing sulphide-oxidizing chemolithotrophs were found only in saline soil SS2 clone library, thus giving the indication of sulphide availability in this soil sample. Barren saline soils favoured diverse photoautotrophic (*Chloroflexi*) and chemolithoautotrophic (*Gammaproteobacteria*) microbial populations. The present study provides basic knowledge about the occurrence of a specific functional bacterial diversity as well as autotrophic potential of bacteria for CO_2_-fixation through the RuBisCO pathway in saline coastal soils. Alternative possible modes and pathways of CO_2_-fixation were not evaluated in this survey but cannot be excluded. However, it will require further investigation including ‘metaproteomics’ [62] which can directly link the microbial community composition to function. Identification of microbial proteins of a given habitat along with their phylogenetic affiliations will provide more comprehensive knowledge of metabolic activities occurring in microbial communities and the possible role of microbial diversity in biogeochemical processes. A better understanding of the resident bacterial communities and their functionalities in the saline barren soils should shed light on the role of barren saline soil as a possible CO_2_ sink.

## Methods

### Site description and sampling

The study was conducted on soil samples of the coastal area of Gujarat, India. Two barren sites and one agricultural field were selected along the sea coast facing the Arabian Sea. Soil samples from the depth of 0 to 10 cm were collected in February 2009. All sampling sites were far away from each other. The three sampling sites were designated as (i) SS1- saline soil samples collected from the barren land away from the sea coast (N 21°35.711’, E 72°16.875’); (ii) SS2- saline soil samples collected from barren land near the sea coast (N 21° 45.402’, E 72° 14.156’); (iii) AS- soil samples collected from the agricultural field (N 20°53.884’, E 70°29.730’). There was no crop in the agricultural field at the time of sample collection. However, farmers grow cotton and groundnut regularly in this field. From each site, soil samples were collected from two different transects and transported to the laboratory in sterile plastic bags. Soil samples were passed through 2 mm pore size sieve to remove rocks and plant materials. Serial dilutions of soil samples were prepared and plated on AT media (Additional file 9: Table S2) for bacterial isolation. DNA extraction was performed immediately from soil samples and the samples were frozen at −20°C for further processing. The pH and salinity were measured using the Seven Easy pH and Conductivity meter (Mettler-Toledo AG, Switzerland) and total soil organic carbon was analyzed by Liqui TOC (Elementar, Germany). CHNS analyzer (Perkin Elmer series ii, 2400) was used for the determination of total carbon, nitrogen and sulphur contents.

### Isolation of bacterial strains

One gram of each soil sample was mixed with 9 mL of normal saline and homogenized for 15 minutes for isolation of *cbbL* gene containing bacterial isolates from the soils. The soil suspension was serially diluted with normal saline to a factor of 10^-6^. Aliquots (100 μL) were spread on AT medium (used for isolation and cultivation of purple non sulphur bacteria) and incubated for three days at 30°C. AT medium [63] was used with some modifications i.e. sodium ascorbate was excluded from the medium and aerobic conditions were used for incubation (Additional file 9: Table S2). Twenty-two morphologically different isolates obtained from three soil samples were streaked on the AT media and incubated for three days at 30°C.

### Amplification and sequencing of *cbbL* and 16S rRNA genes from bacterial isolates

Single colonies from bacterial isolates were inoculated in 5 mL liquid AT medium and incubated at 30°C for 3 days. The cells were centrifuged and used for DNA extraction by Miniprep method [64]. *CbbL* and 16S rRNA genes were amplified using their respective primers and the PCR conditions (Table 3). The amplified and purified PCR products were dried and sent for sequencing (Macrogen Inc., South Korea).

### DNA extraction from soil samples

Genomic DNA was extracted from 0.5 g of soil (from two transects per site) using the fast DNA spin kit for soil (MP Biomedicals, USA) according to the manufacturer’s protocol. To disrupt the cells, the mixture of ceramic and silica beads provided in the kit and two pulses of 30 s and 20 s at speed of 5.5 of the fast prep bead beating instrument were applied. After extraction DNA was quantified and visualized by ethidium bromide-UV detection on an agarose gel.

### Amplification and cloning of *cbbL* and 16S rRNA genes from soil metagenome

The *cbbL* (form IA and IC) and 16S rRNA genes were PCR amplified from total DNA extracted from all the soil samples using same primer sets and PCR conditions as described for bacterial isolates (Table 3). The amplified expected size PCR products were gel purified using the QIAquick gel extraction kit (Qiagen, Hilden, Germany). The purified PCR products were cloned using the TOPO TA cloning kit (Invitrogen, USA) according to the manufacturer’s instructions. The multiple clone libraries for each amplified gene from the soil samples were constructed separately. From each clone library, clones were screened, selected randomly and analyzed for the plasmid containing insert by using the vector specific primers M13F and M13R. The plasmids harbouring the correct size inserts were extracted using alkaline lysis Mini prep method [65] and purified by RNase treatment followed by purification with phenol, chloroform and isoamyl alcohol. The purified plasmids were sent for sequencing to Macrogen Inc. (South Korea). Plasmids were sequenced with the vector specific primers M13F and M13R resulting in sequence lengths of ≈ 1500 bp (16S rRNA genes), ≈800 bp (form IA and form IC *cbbL* genes).

### Alignment and phylogenetic analysis

All sequences were examined for chimeras using the Bellerophon tool [66] with default settings. Seventy five putative chimeric artifacts were removed from further analysis. The BLASTn program was used for retrieval of most similar sequences from GenBank [67]. The 16S rRNA gene sequences were also compared to the current database at the Ribosomal Database Project (RDP) using the RDP sequence match tool [68]. The 16S rRNA gene sequences were assigned to the phylogenetic groups by using RDP classifier [68]. Multiple sequence alignment was performed with Clustal X [69]. Phylogenetic analysis of the *cbbL* and 16S rRNA gene sequences was performed based on the representative OTU (operational taxonomic unit) sequences generated from the Mothur program [36]. Neighbour joining trees for *cbbL* and 16S rRNA nucleotide sequences were constructed by MEGA v.4 with Jukes-Cantor correction model of distance analysis [70]. Bootstrap analysis (1000 replicates) was conducted to test the reliability of phylogenetic tree topology.

### OTU determination and diversity estimation

We used a similarity cut-off of 95% [16] for *cbbL* and 98% [71] for 16S rRNA nucleotide similarity to define an OTU (phylotype) by using Mothur. It uses the furthest neighbour method to assort similar sequences into groups at arbitrary levels of taxonomic similarity. Rarefaction curves, richness estimators and diversity indices were determined with Mothur [36]. Distance matrices were calculated by using the DNADIST program within the PHYLIP software package [72]. We used both the Shannon and Simpson diversity indices and Chao, ACE richness estimators calculated by Mothur to estimate microbial diversity and richness. Percentage of coverage was calculated by Good’s method [73] using the formula C = [1 - (n/N)] x 100, where n is the number of OTUs in a sample represented by one clone (singletons) and N is the total number of sequences in that sample.

### Environmental clustering and comparison of *cbbL/*16S rRNA clone libraries

The differentiation in *cbbL*/16S rRNA clone libraries was analyzed by comparing the coverage levels of the samples, similarities of community membership and the community structure by LIBSHUFF program [74]. It compares homologous and heterologous coverage curves by using the integral form of the Cramer-von Mises statistics and performs multiple pairwise comparisons among a set of libraries. Phylogenetic tree based analysis of community diversity was performed using the UniFrac significance test and the *P* test within UniFrac [75,76]. The rooted phylogenetic tree generated in MEGA along with the environmental labels, was imported into UniFrac. PCA and *P* test analysis was performed within the UniFrac online suite of tools. The *P* test assesses trees for distribution of sequences within the clone libraries according to the environment [77]. All *P* tests reported were also corrected for multiple comparisons (Bonferonni correction).

### Nucleotide sequence accession numbers

The sequences determined in this study have been submitted to GenBank under the accession numbers [GenBank: HQ397346-HQ397353] (form IA *cbbL* sequences from environmental clones), [GenBank: HQ397235-HQ397345, JN202495-JN202546] (form IC *cbbL* sequences from environmental clones), [GenBank: HQ397354-HQ397580] (16S rRNA gene sequences from environmental clones), [GenBank: HQ397588-HQ397594] (form IC *cbbL* sequences from isolates) and [GenBank: HQ397581-HQ397587] (16S rRNA gene sequences from isolates). Representative clone sequences for each OTU from the *cbbL* and 16S rRNA gene libraries were deposited.

## Abbreviations

OTU, Operational taxonomic unit; AS, Agricultural soil; SS1, Saline soil 1; SS2, Saline soil 2.

## Competing interests

The authors declare that they have no competing interests.

## Authors’ contributions

BY: participated in the design of the study, carried out culture independent related experiments, bioinformatics analysis and drafted the manuscript, PS: carried out the culture dependent study, helped in bioinformatics analysis and drafted the manuscript, JK: participated in the study’s design, carried out phylogenetic analysis and drafted the manuscript, BJ: conceived the study and coordination, edited the manuscript and received the funding needed to complete the research. All authors read and approved the final manuscript.

## Supplementary Material

Additional file 1 **Figure S1.** Heat map showing abundance of OTUs in *cbbL-* and 16S rRNA gene clone libraries. The abundance for (a) *cbbL* gene libraries is shown at distance = 0.05 and (b) 16S rRNA gene libraries at distance = 0.02 within the three soil samples. Each row in the heatmap represents a different OTU and the color of the OTU in each group scaled between black and red according to the relative abundance of that OTU within the group. Click here for file

Additional file 2 **Figure S2a.** Phylogenetic analysis of red-like *cbbL* clones from agricultural soil (AS). Bootstrap values are shown as percentages of 1000 bootstrap replicates. The bar indicates 5% estimated sequence divergence. One representative phylotype is shown followed by phylotype number and the number of clones within each phylotype is shown at the end. Clone sequences from AS clone library are coded as ‘BS’. The *cbbL* gene sequences of the isolates are denoted as ‘BSC’. The green-like *cbbL* gene sequence of *Methylococcus capsulatus* was used as outgroup for tree calculations. Click here for file

Additional file 3 **Figure S2b.** Phylogenetic analysis of red-like *cbbL* clones from saline soils (SS1 & SS2) clone libraries. Bootstrap values are shown as percentages of 1000 bootstrap replicates. The bar indicates 5% estimated sequence divergence. One representative phylotype is shown followed by phylotype number and the number of clones within each phylotype is shown at the end. Clone sequences are coded as ‘HS’ (SS1) and ‘R’ (SS2). The *cbbL* gene sequences of the isolates from this study are denoted as ‘HSC’ and ‘RSC’ from SS1 and SS2 respectively. The green-like *cbbL* gene sequence of *Methylococcus capsulatus* was used as outgroup for tree calculations. Click here for file

Additional file 4 **Table S1.** Taxonomic distribution of 16S rDNA clones. The OTUs were generated using a 16S rDNA percent identity value of 98%. Click here for file

Additional file 5 **Figure S3.** Neighbour joining phylogenetic tree of 16S rRNA nucleotide sequences from bacterial isolates. This phylogenetic tree reflecting the relationships of red-like *cbbL* harbouring bacterial isolates with closely related known isolates. 16S rRNA gene sequences of the isolates from this study were denoted as ‘BSCS’ from agricultural soil (AS), ‘HSCS’ from saline soil (SS1) and ‘RSCS’ from saline soil (SS2). *Methanothermobacter autotrophicus* was used as outgroup. The bar indicates 5% estimated sequence divergence. Click here for file

Additional file 6 **Figure S4.** Number of OTUs as a function of total number of sequences. Rarefaction curves for (a) *cbbL* gene libraries at 0.05 distance cut-off and (b) 16S rRNA gene clone libraries at a phylum level distance (0.20) for the expected no of OTUs. Bacterial richness in agricultural soil (AS) and saline soils (SS1 & SS2) is indicated by slopes of the rarefaction curves. Click here for file

Additional file 7 **Figure S5.** Results of selected LIBSHUFF comparisons. (I) 16S rRNA libraries (a1) AS (X) to SS1 (Y), (a2) libraries AS (X) to SS2 (Y) and (a3) libraries SS1 (X) to SS2 (Y). (II) *CbbL* libraries (b1) ASC (X) to SS1C (Y), (b2) libraries ASC (X) to SSC2 (Y) and (b3) libraries SS1C (X) to SS2C(Y). Agricultural soil is denoted as ‘AS’ while as saline soils are denoted as ‘SS1 & SS2’. Click here for file

Additional file 8 **Figure S6.** Venn diagrams showing overall overlap of representative genera. Venn diagrams representing the observed overlap of OTUs for (a) *cbbL* gene libraries (distance = 0.05) and (b) 16S rRNA gene libraries (distance = 0.02). The values in the diagram represent the number of genera that were taxonomically classified. Click here for file

Additional file 9 **Table S2.** Composition of AT media (Imhoff). Click here for file
