# Supplementary figures and images for: Comparative molecular analysis of chemolithoautotrophic bacterial diversity and community structure from coastal saline soils, Gujarat, India
Source: BMC Microbiol. 2012 Jul 26;12:150. doi: 10.1186/1471-2180-12-150 (PMC3438102; doi:10.1186/1471-2180-12-150)

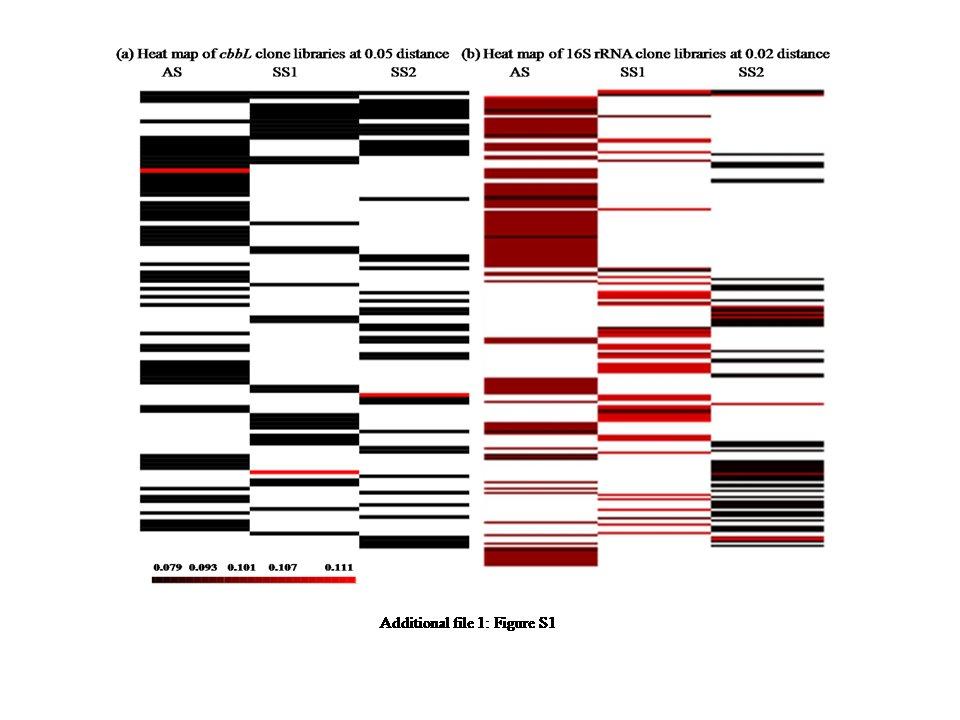

Supplement: Additional file 1 — Figure S1. Heat map showing abundance of OTUs in cbbL- and 16S rRNA gene clone libraries. The abundance for (a) cbbL gene libraries is shown at distance = 0.05 and (b) 16S rRNA gene libraries at distance = 0.02 within the three soil samples. Each row in the heatmap represents a different OTU and the color of the OTU in each group scaled between black and red according to the relative abundance of that OTU within the group. [file 1471-2180-12-150-S1.jpeg]

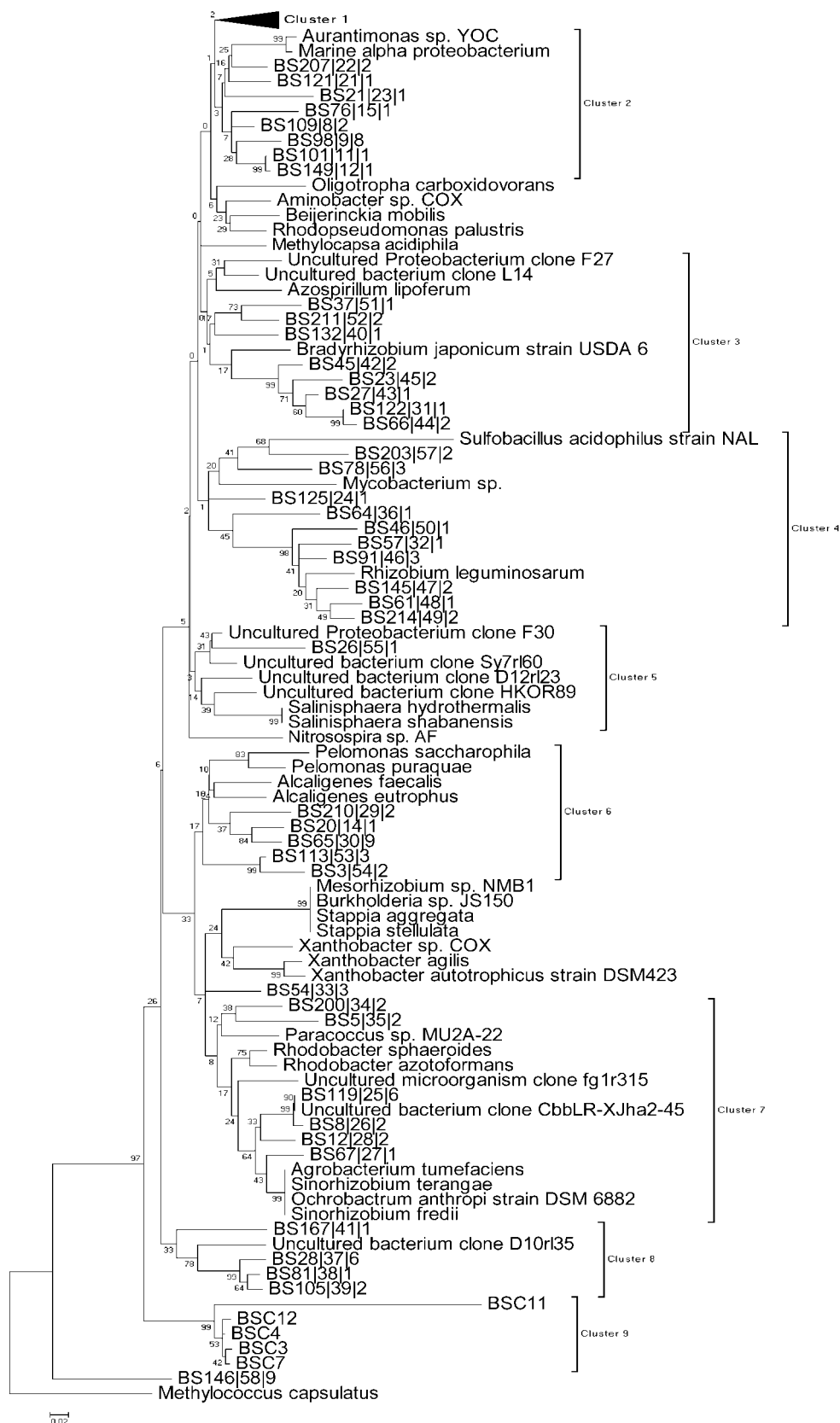

Additional file 2: Figure S2a

Supplement: Additional file 2 — Figure S2a. Phylogenetic analysis of red-like cbbL clones from agricultural soil (AS). Bootstrap values are shown as percentages of 1000 bootstrap replicates. The bar indicates 5% estimated sequence divergence. One representative phylotype is shown followed by phylotype number and the number of clones within each phylotype is shown at the end. Clone sequences from AS clone library are coded as ‘BS’. The cbbL gene sequences of the isolates are denoted as ‘BSC’. The green-like cbbL gene sequence of Methylococcus capsulatus was used as outgroup for tree calculations. [file 1471-2180-12-150-S2.pdf]

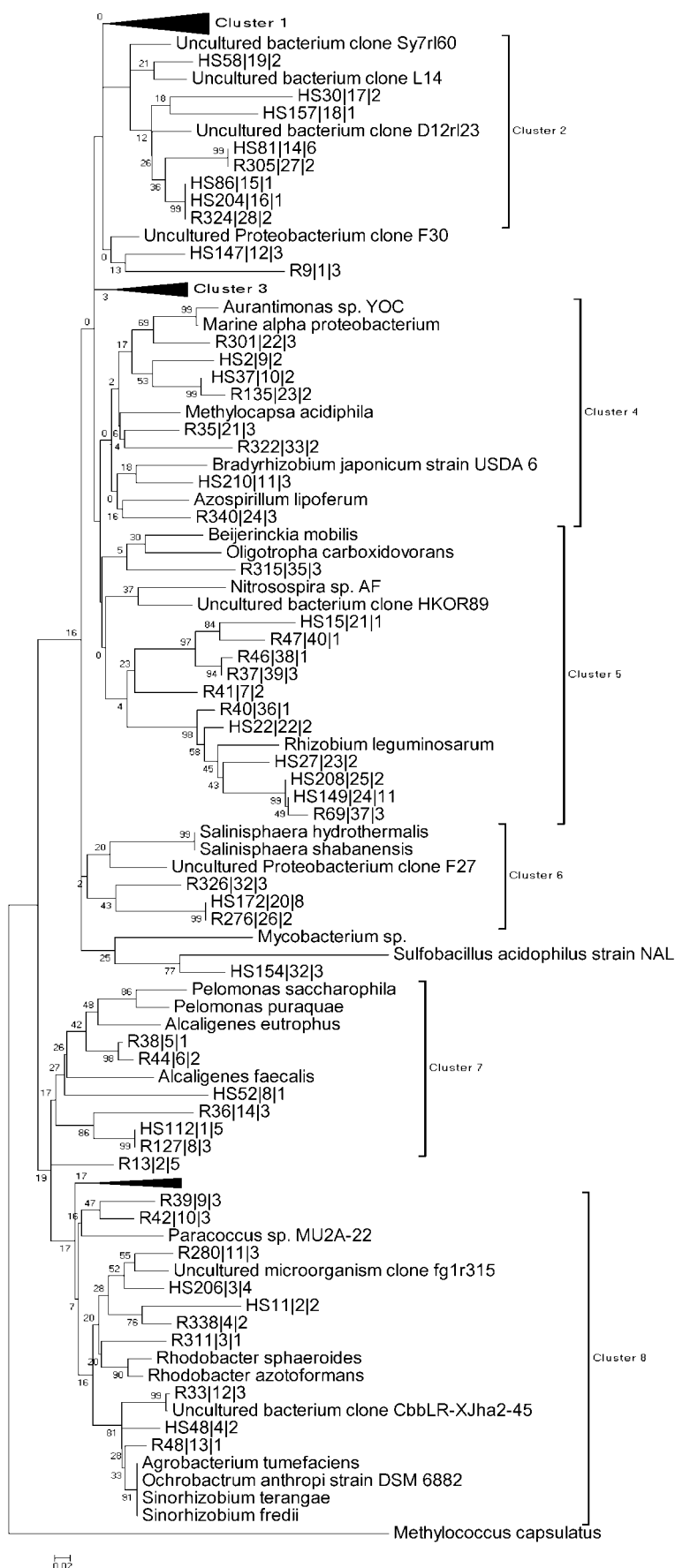

Additional file 3: Figure S2b

Supplement: Additional file 3 — Figure S2b. Phylogenetic analysis of red-like cbbL clones from saline soils (SS1 & SS2) clone libraries. Bootstrap values are shown as percentages of 1000 bootstrap replicates. The bar indicates 5% estimated sequence divergence. One representative phylotype is shown followed by phylotype number and the number of clones within each phylotype is shown at the end. Clone sequences are coded as ‘HS’ (SS1) and ‘R’ (SS2). The cbbL gene sequences of the isolates from this study are denoted as ‘HSC’ and ‘RSC’ from SS1 and SS2 respectively. The green-like cbbL gene sequence of Methylococcus capsulatus was used as outgroup for tree calculations. [file 1471-2180-12-150-S3.pdf]

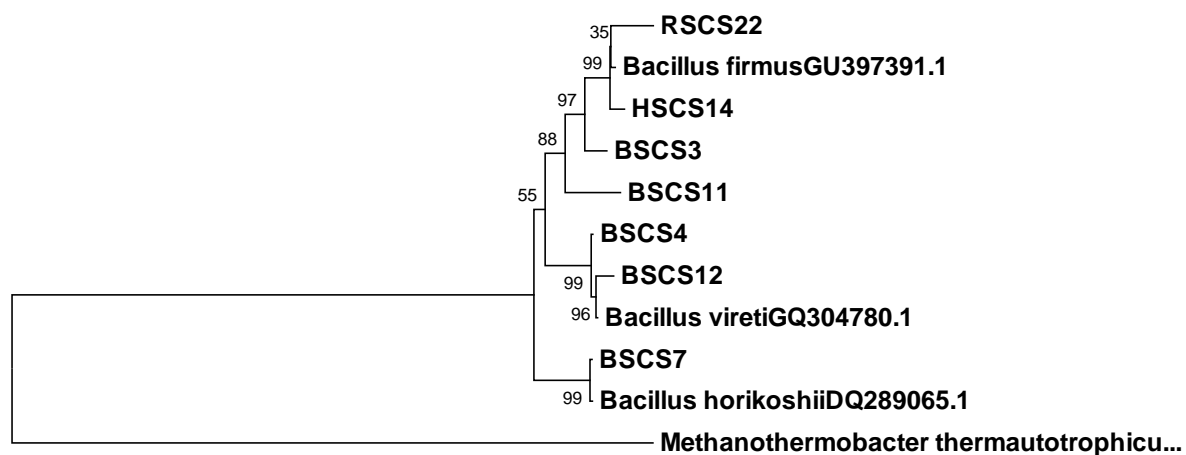

Additional file 5: Figure S3

Supplement: Additional file 5 — Figure S3. Neighbour joining phylogenetic tree of 16S rRNA nucleotide sequences from bacterial isolates. This phylogenetic tree reflecting the relationships of red-like cbbL harbouring bacterial isolates with closely related known isolates. 16S rRNA gene sequences of the isolates from this study were denoted as ‘BSCS’ from agricultural soil (AS), ‘HSCS’ from saline soil (SS1) and ‘RSCS’ from saline soil (SS2). Methanothermobacter autotrophicus was used as outgroup. The bar indicates 5% estimated sequence divergence. [file 1471-2180-12-150-S5.pdf]

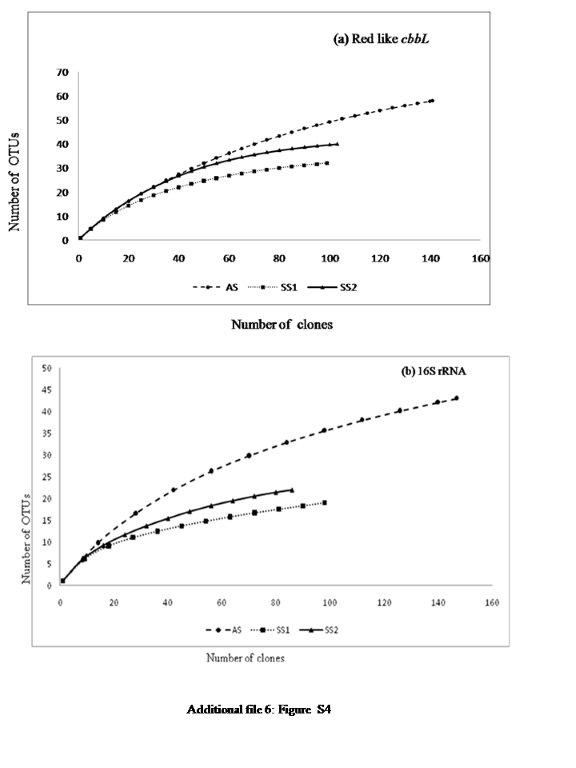

Supplement: Additional file 6 — Figure S4. Number of OTUs as a function of total number of sequences. Rarefaction curves for (a) cbbL gene libraries at 0.05 distance cut-off and (b) 16S rRNA gene clone libraries at a phylum level distance (0.20) for the expected no of OTUs. Bacterial richness in agricultural soil (AS) and saline soils (SS1 & SS2) is indicated by slopes of the rarefaction curves. [file 1471-2180-12-150-S6.jpeg]

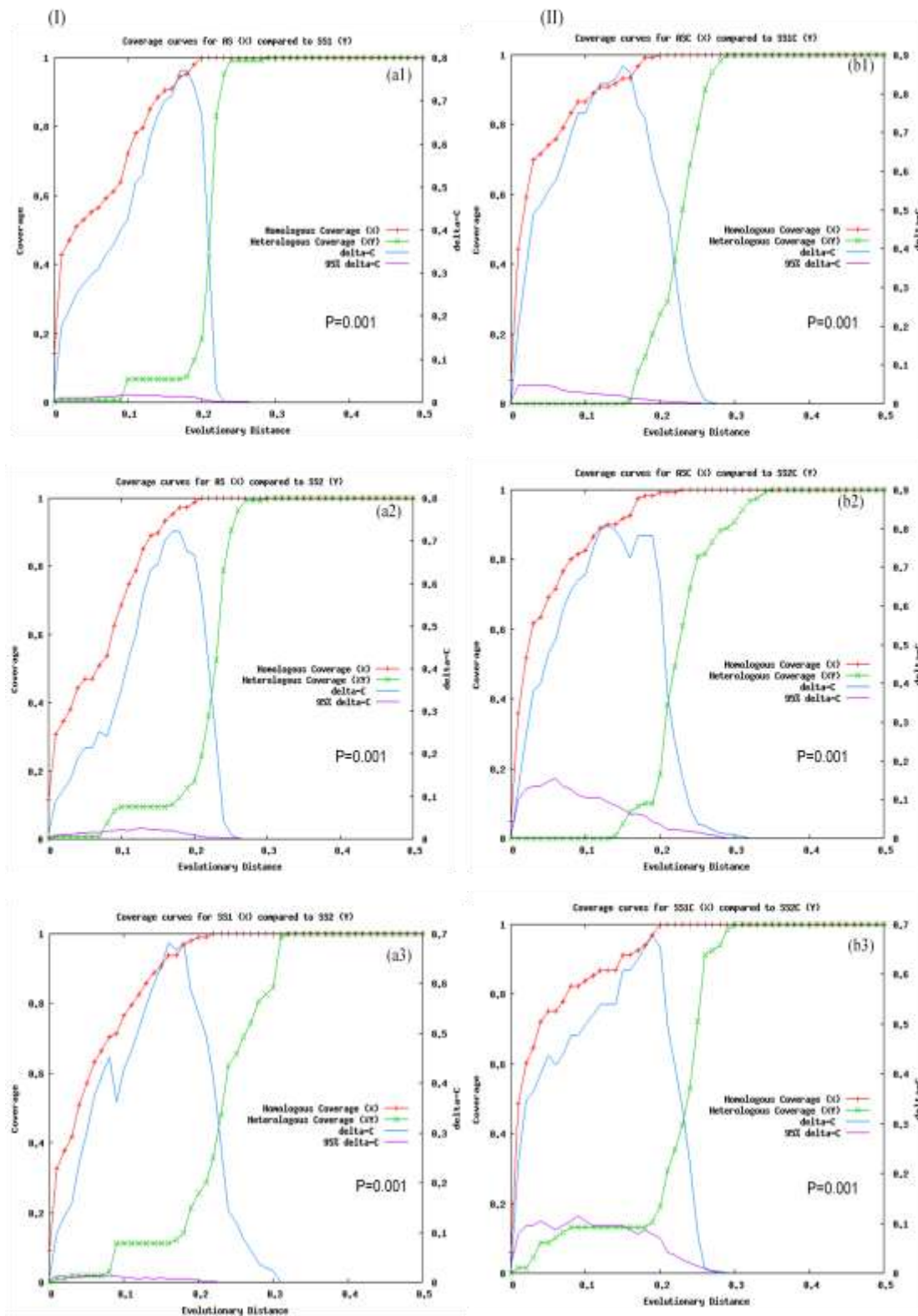

Additional file 7: Figure S5

Supplement: Additional file 7 — Figure S5. Results of selected LIBSHUFF comparisons. (I) 16S rRNA libraries (a1) AS (X) to SS1 (Y), (a2) libraries AS (X) to SS2 (Y) and (a3) libraries SS1 (X) to SS2 (Y). (II) CbbL libraries (b1) ASC (X) to SS1C (Y), (b2) libraries ASC (X) to SSC2 (Y) and (b3) libraries SS1C (X) to SS2C(Y). Agricultural soil is denoted as ‘AS’ while as saline soils are denoted as ‘SS1 & SS2’. [file 1471-2180-12-150-S7.pdf]

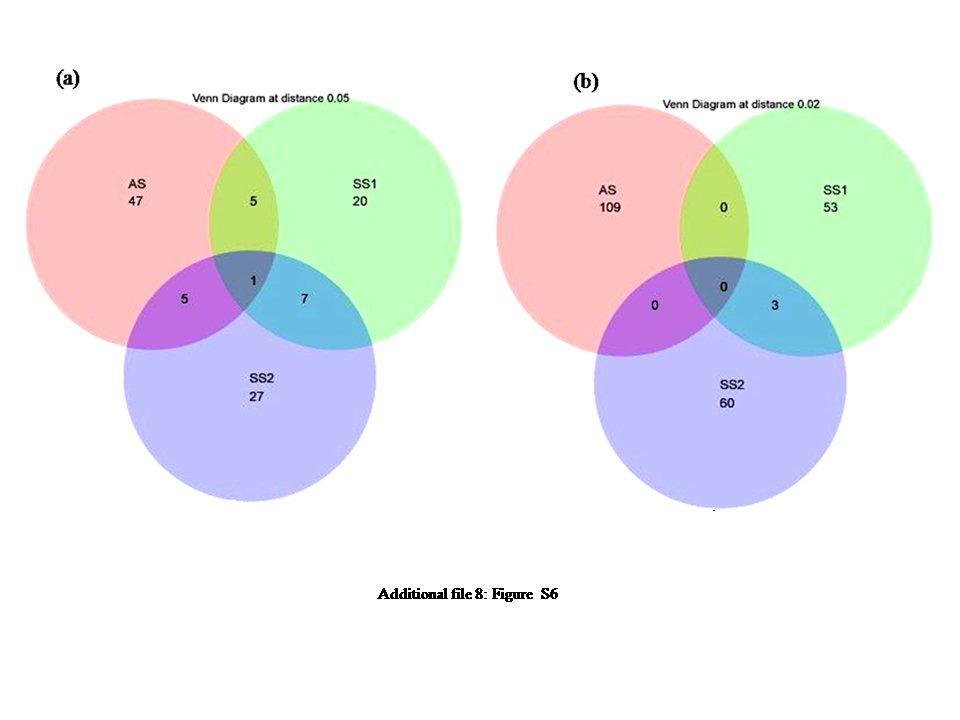

Supplement: Additional file 8 — Figure S6. Venn diagrams showing overall overlap of representative genera. Venn diagrams representing the observed overlap of OTUs for (a) cbbL gene libraries (distance = 0.05) and (b) 16S rRNA gene libraries (distance = 0.02). The values in the diagram represent the number of genera that were taxonomically classified. [file 1471-2180-12-150-S8.jpeg]
